# An Image Dehazing Algorithm for Underground Coal Mines Based on gUNet

**DOI:** 10.3390/s24113422

**Published:** 2024-05-26

**Authors:** Feng Tian, Lishuo Gao, Jing Zhang

**Affiliations:** 1 College of Communication and Information Technology, Xi’an University of Science and Technology, Xi’an 710600, China; tianfeng@xust.edu.cn; 2 Xi’an Key Laboratory of Network Convergence Communication, Xi’an University of Science and Technology, Xi’an 710054, China; 3 College of Computer Science and Technology, Xi’an University of Science and Technology, Xi’an 710600, China

**Keywords:** underground coal mines, image dehazing, U-Net, DSConv, residual attention convolution, CA, fusion loss function

## Abstract

Aiming at the problems of incomplete dehazing, color distortion, and loss of detail and edge information encountered by existing algorithms when processing images of underground coal mines, an image dehazing algorithm for underground coal mines, named CAB CA DSConv Fusion gUNet (CCDF-gUNet), is proposed. First, Dynamic Snake Convolution (DSConv) is introduced to replace traditional convolutions, enhancing the feature extraction capability. Second, residual attention convolution blocks are constructed to simultaneously focus on both local and global information in images. Additionally, the Coordinate Attention (CA) module is utilized to learn the coordinate information of features so that the model can better capture the key information in images. Furthermore, to simultaneously focus on the detail and structural consistency of images, a fusion loss function is introduced. Finally, based on the test verification of the public dataset Haze-4K, the Peak Signal-to-Noise Ratio (PSNR), Structural Similarity (SSIM), and Mean Squared Error (MSE) are 30.72 dB, 0.976, and 55.04, respectively, and on a self-made underground coal mine dataset, they are 31.18 dB, 0.971, and 49.66, respectively. The experimental results show that the algorithm performs well in dehazing, effectively avoids color distortion, and retains image details and edge information, providing some theoretical references for image processing in coal mine surveillance videos.

## 1. Introduction

The coal mine video surveillance system is a crucial equipment for investigating potential safety hazards for personnel underground. However, due to insufficient illumination in underground coal mines, the mining process generates a significant amount of dust and water haze. The coal dust particles and haze cause light scattering and absorption, resulting in the video surveillance system capturing images that exhibit noticeable dust and haze effects, such as blurriness, low contrast, and loss of details. These issues severely affect the image quality, which not only hinder the performance of subsequent advanced computer vision tasks such as object recognition and tracking but also pose severe challenges to the productivity and safety of miners. Therefore, research on image dehazing algorithms for underground coal mines is of great significance.

Currently, there are roughly three categories of dehazing algorithms: dehazing algorithms based on image enhancement, dehazing algorithms based on image restoration, and dehazing algorithms based on deep learning. Among them, the dehazing algorithms based on image enhancement primarily reduce the influence of haze on images by enhancing image contrast and color saturation. Although these algorithms are simple in principle and fast in processing, they are sensitive to changes in environmental lighting, which may lead to significant performance variations under different lighting conditions. They are not suitable for low-light environments such as underground coal mines. Additionally, these methods do not consider the principles of image degradation, resulting in the loss of pixel information, and therefore, the effects are often unsatisfactory.

The dehazing algorithms based on image restoration, which are mostly based on the Atmospheric Scattering Model (ASM) [1,2], achieve dehazing by estimating the transmission map and atmospheric light [3,4,5,6,7,8]. Compared with the dehazing algorithms based on image enhancement, these consider the imaging principle of haze images and improve dehazing performance. However, they tend to be computationally intensive when estimating intermediate parameters, which affects the efficiency of dehazing. Additionally, the accumulated errors during parameter estimation can lead to incomplete dehazing and color distortion in the processed images. For example, He et al. [3] proposed the Dark Channel Prior (DCP) dehazing algorithm, improving dehazing to some extent but being prone to errors in estimating atmospheric light, leading to color distortion. Wang et al. [7] proposed an image dehazing algorithm of underground coal mines based on adaptive dual-channel priors, which effectively removes dust and haze in images, enhances image detail information, and reduces runtime. Cao et al. [8] introduced an image dehazing algorithm of underground coal mines based on a boundary constraint, achieving dehazing by integrating the DCP algorithm with boundary-constrained and context-regularized algorithms. In summary, while traditional dehazing methods are fast and relatively simple to implement, their dehazing effects are not ideal.

With the development of deep learning, an increasing number of researchers are applying it to the field of image dehazing. Early dehazing algorithms based on deep learning were mostly founded on the ASM, utilizing Convolutional Neural Networks (CNNs) to estimate the parameters of the ASM [9,10,11,12,13,14,15]. For instance, Cai et al. [10] designed an end-to-end system to estimate the transmission map of haze images and then restored the haze-free images based on the ASM. However, this model primarily considers single light source scenarios and is less effective in outdoor dehazing contexts. Li et al. [11] modified the formula of the ASM, combining the transmission map and atmospheric light into a single parameter, and then built a lightweight network (All-in-One Dehazing Network, AOD-Net) for efficient parameter estimation. Ullah et al. [13] built upon this modified formula to propose LD-Net (Light-DehazeNet), a computationally efficient lightweight CNN for parameter estimation, and introduced a new method to avoid color distortion. Fan et al. [15] proposed an efficient dehazing method combining lightweight CNN and color visibility restoration, which improves the dehazing effect and visual quality of coal mine underground images. However, the model’s complexity and computational cost are relatively high. In summary, while these deep learning algorithms based on the ASM have improved dehazing effects to varying degrees, they still rely on the estimation of a transmission map and atmospheric light, thus making it difficult to avoid inaccuracies in parameter estimation, leading to the inability to generate high-precision images.

In recent years, some dehazing algorithms based on CNNs have gradually moved away from relying on ASM, eliminating the need to estimate a transmission map and atmospheric light. Instead, these models are trained using datasets to generate haze-free images through the design of different neural networks [16,17,18,19,20,21,22,23,24,25,26,27]. For example, Qin et al. [18] designed the Feature Fusion Attention Network (FFA-Net) for dehazing, integrating feature attention mechanisms to improve the representation of CNNs. However, the resulting images often lack clarity in details and edges. Dong et al. [19] designed the Multi-Scale Boosted Dehazing Network (MSBDN) based on U-Net [28,29,30], featuring dense feature fusion, progressively restoring haze-free images through an enhanced decoder. Song et al. [20] introduced a gUNet model based on U-Net, utilizing a gating mechanism instead of pixel attention modules and non-linear activation functions to model the spatially varying transmission mapping, which significantly improved dehazing effects, but the generated images suffered from color distortion, detail loss, and edge information omission. Furthermore, Song et al. [21] developed the DehazeFormer model based on the Swin Transformer [31], with various improvements, including enhanced normalization layers, activation functions, and spatial information aggregation. Wu et al. [26] significantly enhanced the performance of dehazing real-world images by introducing high-quality priors (HQPs) and a phenomenological degradation pipeline. Nevertheless, this method still has limitations when dealing with non-uniform and extremely dense haze. Cong et al. [27] developed a spatial and frequency domain-aware semi-supervised nighttime dehazing network (SFSNiD), which significantly improved nighttime image dehazing through a spatial and frequency domain information interaction (SFII) module and a semi-supervised retraining strategy. Although such algorithms substantially enhance the quality of generated images, their adaptability to new samples is weak, resulting in poor generalization capabilities. Due to insufficient illumination and severe dust and water haze in underground coal mines, the captured video images are blurry with poor detail. These methods have not been optimized for the lack of detailed information in coal mine underground scenes, leading to unsatisfactory dehazing performance in such environments.

With the significant advances in deep-learning-based dehazing algorithms, their application in various complex environments has gradually attracted attention. However, research on image dehazing specifically for coal mine underground environments remains limited. In response, this paper proposes a novel image dehazing algorithm for underground coal mines, named CCDF-gUNet. Initially, the algorithm employs a lightweight U-Net model with an encoder–decoder structure as its base architecture for extracting multi-scale feature information from images, and Dynamic Snake Convolution (DSConv) is introduced to replace traditional convolutions, enhancing the feature extraction capability. Subsequently, to enhance the model’s ability to perceive important features in images, residual attention convolutional blocks and CA modules are constructed. Additionally, to better cope with the complex environments in a mine, a fusion loss function is introduced. This loss function integrates the advantages of the MS-SSIM loss and the L1 loss, which improves the perceptual quality and structural consistency of images while preserving image details. Through this research, we aim to provide a reliable image processing method for the coal mining industry, enhancing the productivity and safety of miners and promoting sustainable development in the coal mining industry.

The primary contributions of this paper can be summarized as follows:

(a) The design of residual attention convolutional blocks and the introduction of a CA module, embedding CAB into residual attention convolution blocks to simultaneously focus on both local and global information in the image, as well as using CA modules to learn feature coordinate information, so that the model can better capture key information in images.
(b) The introduction of DSConv to replace the first 3 × 3 convolution layer in the encoder part of the network structure enhances the feature extraction capability by adaptively adjusting the shape and position of the convolution kernel.
(c) The introduction of a fusion loss function combining the MS-SSIM loss and the L1 loss, focusing on both the detail and the structural consistency of images, to optimize the pixel-level accuracy and visual quality of restored images in mine environments.
(d) Due to the lack of publicly available datasets on underground coal mines, a large collection of underground coal mine images and surveillance videos from various mine and time periods is gathered, covering typical underground scenes like conveyor belts, mining faces, and tunnels, ensuring the diversity and representativeness of the dataset.
(e) The proposed CCDF-gUNet dehazing algorithm is tested and validated on the public dataset Haze-4K and a self-made underground coal mine dataset, demonstrating its effectiveness in real mining environments.

The rest of the paper is organized as follows: Section 2 discusses related work, analyzing the progress and pros and cons of attention mechanisms, CNNs, and loss functions. Section 3 consists of three parts, namely, the theoretical basis of attention mechanisms, CNNs, and the fusion loss function. Section 4 describes the experimental setup, including the datasets, experimental configurations, and evaluation metrics. It then provides comparative experiments for attention mechanisms, CNNs, and loss functions, followed by comparisons with the state-of-art dehazing algorithms and ablation studies. Section 5 provides a systematic conclusion.

## 2. Related Work

### 2.1. Attention Mechanism

In recent years, the attention mechanism has been widely used in the field of computer vision. By weighting input features, it can concentrate the model’s focus on the most crucial areas, thereby enhancing performance and effectiveness. Due to its excellent plug-and-play convenience, the attention mechanism has gradually been applied to various deep learning models in the field of image processing.

Firstly, Hu et al. [32] proposed the channel attention mechanism Squeeze-and-Excitation Network (SE-Net), which learns adaptive channel weights to make the model pay more attention to useful channel information. However, it overlooks the importance of the spatial dimension and cannot fully capture the spatial features in images. To address this issue, the Convolutional Block Attention Module (CBAM) [33] was introduced. CBAM combines channel attention and spatial attention, considering both the inter-channel relationships and the importance of spatial locations, thereby capturing essential features in images more comprehensively. Although CBAM has made some progress in capturing spatial and channel features, there is still room for improvement in capturing fine-grained features and edge details. Counterfactual Attention Learning (CAL) [34] introduced a novel approach by generating virtual counterfactual samples to distinguish between truly important features and noise, helping the model better understand important regions and features in images. This not only enhances the model’s sensitivity to fine-grained features but also effectively suppresses background noise interference. However, this method increases computational complexity and is less effective when handling large-scale data. Subsequently, the Cross Attention Block (CAB) [35] improved the focus on important regions and details in images by applying an attention inner patch and between patches, capturing both local and global information. This method reduces computational complexity while better highlighting significant regions and edge details in images. To further optimize the attention mechanism, Coordinate Attention (CA) [36] was proposed. CA embeds spatial position information into channel attention, more effectively capturing positional information and channel relationships to enhance the network’s feature representation. Compared with traditional attention mechanisms, CA not only considers inter-channel relationships but also utilizes positional information to generate more selective attention feature maps, enhancing the model’s ability to perceive critical regions in images.

By combining CAB and CA modules, their advantages can be fully leveraged, allowing the model to excel in capturing local, global, and spatial information. Specifically, this combination enables the model to more comprehensively capture the contextual information of images, focus more on crucial regions and edge details, emphasize the importance of positional information, and improve the network’s ability to perceive key areas in images. This significantly enhances the dehazing effect and image quality.

### 2.2. Convolutional Neural Networks

Convolutional Neural Networks (CNNs) have been widely utilized in the realms of image processing and computer vision, particularly in tasks such as image dehazing, image segmentation, and object detection. With the continuous advancement of deep learning in recent years, traditional convolution methods have undergone various improvements to enhance their feature extraction capabilities and computational efficiency. Traditional CNNs employ convolutional kernels of fixed shapes to extract features from images, achieving remarkable results. However, the fixed shape of convolutional kernels poses certain limitations when dealing with complex edges and detailed information. In image dehazing tasks, the restoration of details is crucial for dehazing effectiveness, driving continuous improvement and optimization of convolution methods.

Dynamic convolution (DyConv) [37] addresses this issue by dynamically generating convolutional kernels for each input sample, thus adapting to variations in the input data. This approach captures more details and features, significantly improving performance in image processing tasks. However, DyConv demands substantial computational resources due to its increased computational complexity. Ghost convolution (GhostConv) [38] reduces computational load by generating redundant feature maps, thereby lowering computational costs while maintaining high feature extraction capabilities, though it still faces limitations in handling complex image structures. Depthwise separable convolution (DSC) [39,40] decomposes standard convolution into depthwise convolution and pointwise convolution, substantially reducing the computational load and parameter count. This method is widely employed in lightweight network architectures such as MobileNet, becoming a crucial technology for enhancing network computational efficiency. Nonetheless, it has shortcomings in capturing global features and long-range dependencies.To address these issues, Dynamic Snake Convolution (DSConv) [41] emerges as a novel convolution method designed to better capture fine shapes and edge information in images. DSConv introduces a dynamic adjustment mechanism that allows convolutional kernels to adaptively adjust based on the local features of the input image, thereby more effectively restoring edges and details obscured by haze. This demonstrates its significant potential in image dehazing tasks.

### 2.3. Loss Function

The L1 loss function, also called the absolute value loss function, calculates the absolute difference between the predicted and the real values, disregarding the direction of the difference and focusing only on its magnitude. This makes the L1 loss function more robust in dealing with noise and outliers, maintaining image stability. However, there are some limitations to the L1 loss function. For instance, it only considers the pixel-level differences and disregards the human eye’s perception of structure and texture. Moreover, it lacks structural preservation, treating each pixel location equally without considering the structural information of images. To better simulate the human eye’s perception of image quality and enhance model performance, Wang et al. [42] introduced the Structural Similarity (SSIM) loss function, which introduces luminance, contrast, and structural factors for better image smoothness retention. By incorporating the Structural Similarity Index, it better simulates the human perception of image quality. Wang et al. [43] also proposed the Multi-Scale Structural Similarity (MS-SSIM) loss function, an enhancement of the SSIM loss function, which provides a more comprehensive assessment of image quality by comparing Structural Similarity at multiple scales. It introduces features of multiple scales to better capture the details and structure of images more effectively, enabling the MS-SSIM loss function to evaluate images more comprehensively, especially those with complex textures and rich details. Compared with SSIM, MS-SSIM better captures multi-scale features and perceptual details of images, thereby enhancing model performance. Consequently, we present a new loss function, the fusion loss function, which combines the strengths of MS-SSIM and the L1 loss, preserving the image detail while also enhancing the perceptual quality and structural consistency of images.

## 3. Methods

**Figure 1:**
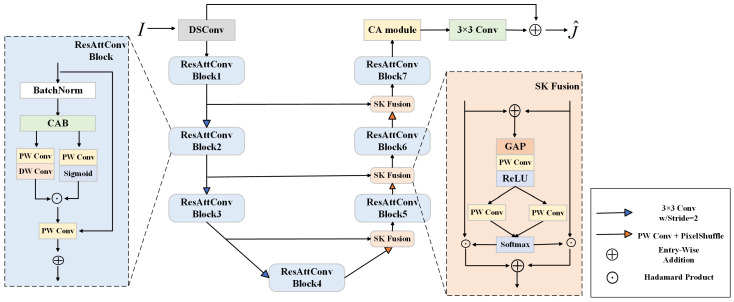
Overall structure of the dehazing network, using lightweight U-Net as the basic architecture. The process involves inputting an image from underground coal mines into the network and obtaining a clear, haze-free image through operations such as downsampling, upsampling, and feature fusion.

The proposed image dehazing network for underground coal mines, CCF-gUNet, adopts an encoder–decoder framework [44]. The overall structure of the model is shown in Figure 1.

Initially, the network employs a lightweight U-Net model as its basic architecture for multi-scale information extraction and utilizes depthwise separable convolution layers to aggregate spatial information, significantly reducing the number of parameters. Additionally, DSConv is introduced to replace the first 3 × 3 convolution layer in the encoder part, enhancing the feature extraction capability by adaptively adjusting the shape and position of the convolution kernel. Subsequently, a color correction module composed of residual attention convolution blocks embedded with CAB is constructed, adaptively adjusting the weights of features to increase the adaptability of the network and generalization ability. Additionally, the model uses the SK fusion module to dynamically integrate feature maps from different pathways, better adapting to the effects of varying degrees of haze. Moreover, to focus on image details and edge areas, a CA module is introduced following the feature fusion to adjust the attention weights at individual positions based on location information, which improves the robustness of the algorithm. Finally, given a pair of images {I(x),J(x)}, the network predicts the global residual $R\left(x\right)=\hat{J}\left(x\right)-I\left(x\right)$, and the model is trained using the fusion loss function, making it more suitable for the real environment of underground coal mines.

### 3.1. Attention Modules

To more effectively capture spatial contextual information and further enhance the dehazing performance in underground coal mines, this paper introduces residual attention convolutional blocks by incorporating the CAB within the residual convolutional blocks. Additionally, the CA module is incorporated following the feature fusion. The CAB captures both local and global information by applying an attention inner patch and between patches, thereby increasing the focus on important regions and details in images. This facilitates a more accurate restoration of image details and colors. The CA module, on the other hand, is more concerned with important areas and edge detail information in images, emphasizing the significance of positional information, thus enhancing the network’s perception of key areas in images.

#### 3.1.1. Residual Attention Convolution Block

**Figure 2:**
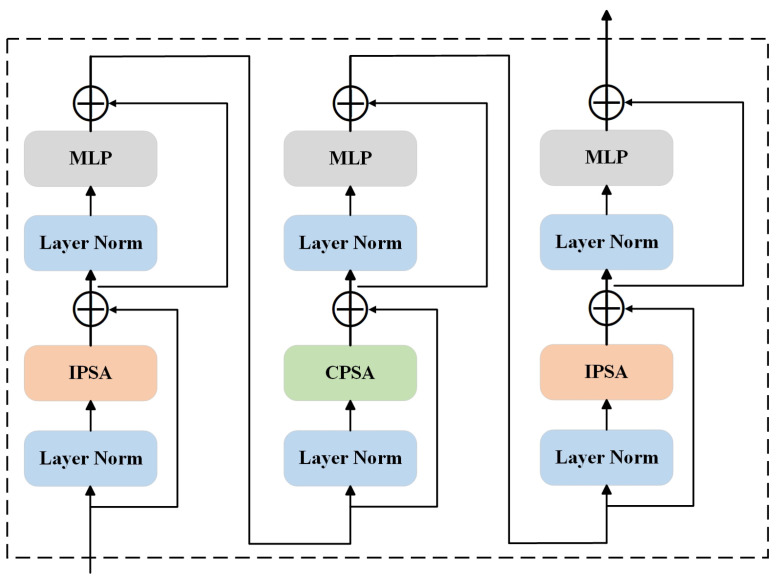
Cross Attention Block (CAB) network structure. CAB forms an effective attention module capable of capturing both local and global information by stacking Inner-Patch Self-Attention Block (IPSA) and Cross-Patch Self-Attention Block (CPSA) modules, along with Layer Normalization (LN), Multi-Layer Perceptron (MLP), and shortcut.

In this paper, CAB is introduced into residual convolutional blocks, aiming to enhance the model’s representational capability when processing images by learning the relationship between local detail and global contextual information. CAB comprises two Inner-Patch Self-Attention Blocks (IPSA) and one Cross-Patch Self-Attention Block (CPSA) on a single-channel feature map, as illustrated in Figure 2.

In computer vision, each pixel requires specific channels to represent its different semantic features, and directly applying self-attention mechanisms to the entire image would result in a very high computational load. Inspired by the local feature extraction capability of CNNs, IPSA captures local information by applying the self-attention mechanism inner patch of the image, significantly reducing computational complexity.

The computational complexity of traditional Multi-Head Self-Attention (MSA) increases quadratically with the resolution of the input image. The formula is as follows:

(1) $$\begin{array}{c} FLOPs_{MSA}=4HWC^{2}+2H^{2}W^{2}C \end{array}$$

After introducing IPSA, the computational complexity is significantly reduced. The complexity of IPSA is linearly related to the patch size. The formula is as follows:

(2) $$\begin{array}{c} FLOPs_{IPSA}=4HWC^{2}+2N^{2}HWC \end{array}$$

where *N* represents the patch size. Assuming *H*, *W* = 56, *C* = 96, and *N* = 7, the FLOPs of traditional MSA is approximately 2.0 G, while the FLOPs of IPSA is about 0.15 G, showing a significant reduction in computational load.

While IPSA is effective at capturing local information, considering only local information is insufficient; global information extraction is equally important. In traditional CNNs, the receptive field is usually expanded by stacking convolutional kernels to capture a broader range of contextual information. However, Transformers inherently have the ability to capture global information. CPSA captures global information by applying the self-attention mechanism on a single-channel feature map, treating each channel as an individual feature map, which is then divided into multiple patches with self-attention applied between these patches. This approach is analogous to the idea of depthwise separable convolutions, significantly reducing computational complexity while retaining the ability to capture global information.

The computational complexity of CPSA is related to the patch size and feature map dimensions. The formula is as follows:

(3) $$\begin{array}{c} FLOPs_{CPSA}=4N^{2}HWC+2{\left(HW/N\right)}^{2}C \end{array}$$

where *N* is the patch size, and *H* and *W* represent the height and width of the feature map, respectively. Compared with MSA, the computational complexity of CPSA is significantly reduced. Assuming *H*, *W* = 56, *C* = 96, and *N* = 7, the FLOPs of MSA is approximately 2.0 G, while the FLOPs of CPSA is about 0.1 G.

**Figure 3:**
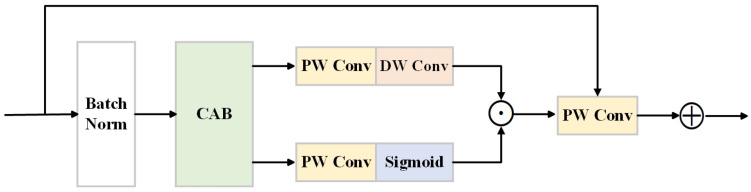
Structure of residual attention convolution block. It combines Batch Normalization, CAB, Pointwise Convolutions (PWConvs), and Depthwise Convolutions (DWConvs) with a sigmoid activation for channel-wise attention, enhancing feature selection before the final pointwise convolution.

In the network structure presented in this paper, we constructed residual attention convolutional blocks as color restoration modules to enhance the network’s feature selection capability, as shown in Figure 3. CAB is introduced into each residual convolutional block, allowing the network to dynamically adjust feature weights after convolution operation, aiding in the accurate restoration of true colors during the dehazing process and avoiding color distortion. By combining CPSA with IPSA, the model ensures the capture of both local detail information and global contextual information, providing a solid foundation for subsequent feature processing and information fusion. This structure design fully leverages the advantages of both Transformers and CNNs, enhancing the model’s feature representation capability while maintaining computational efficiency.

#### 3.1.2. CA Module

**Figure 4:**
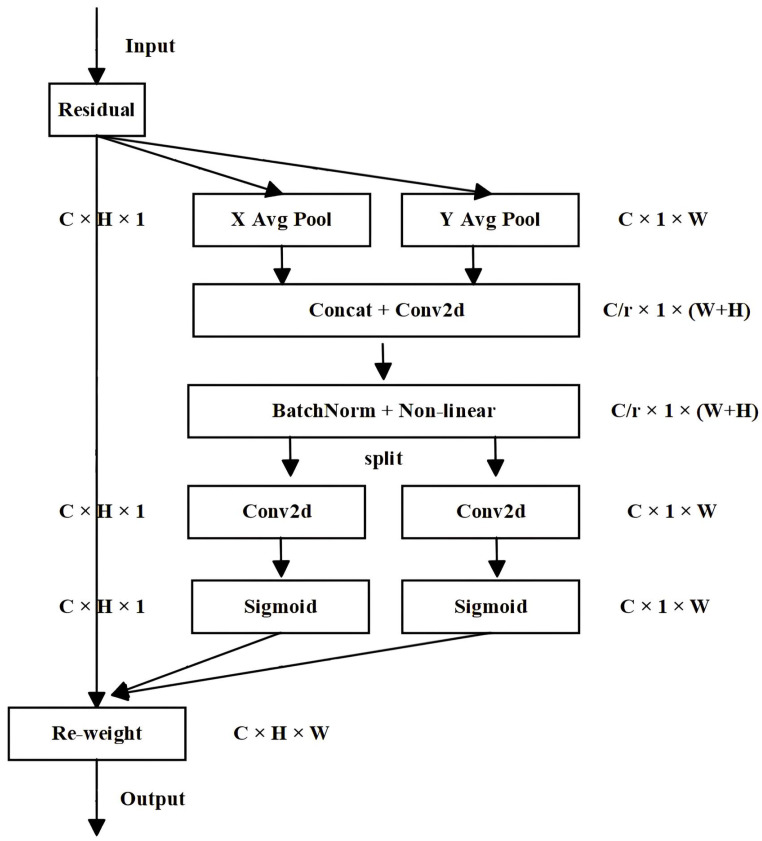
Flowchart of the Coordinate Attention(CA) module. The operations of the CA module are divided into two steps: coordinate information embedding and Coordinate Attention generation.

The Coordinate Attention (CA) module is also introduced in this paper. It is a lightweight attention mechanism module that considers channel and spatial aspects in parallel, embedding positional information into channel attention, aiming to further enhance the performance of the underground coal mines’ dehazing network. Traditional attention mechanisms often lack the capability to capture long-distance relationships and ignore the spatial positional information between pixels. However, for the complex environment in underground coal mines, the positional relationship between pixels is critical to the success of the dehazing task. By incorporating the CA module, the paper can better model spatial relationships between pixels, thereby improving its perception of different positions in images. The overall flowchart of the CA module is illustrated in Figure 4.

As can be seen from the figure, the input feature map is divided into two directions, height and width, for global average pooling, to obtain feature maps in these two directions, as shown in Equations (4) and (5).

(4) $$z_{c}^{h}\left(h\right)=\frac{1}{W}\sum_{0\leq i<W}x_{c}\left(h,i\right)$$

(5) $$z_{c}^{w}\left(w\right)=\frac{1}{H}\sum_{0\leq j<H}x_{c}\left(j,w\right)$$

Subsequently, the feature maps obtained from the two directions of the global receptive are concatenated. Then, they pass through a shared convolution module with 1×1 convolution kernels to reduce their directions to C/r of the original size. After that, the batch-normalized feature map $F_{1}$ is fed into a sigmoid activation function to obtain a feature map *f* of size C/r×1×(W+H), as shown in Equation (6).

(6) $$f=\delta \left(F_{1}\left(\left[z^{h},z^{w}\right]\right)\right)$$

Next, the feature map *f* undergoes 1×1 convolution according to the original height and width, obtaining feature maps with the same number of channels as the original, denoted as $F_{h}$ and $F_{w}$. These are then passed through the sigmoid activation function to separately acquire attention weights for the feature map in the two directions $g^{h}$ and $g^{w}$, respectively, as illustrated in Equations (7) and (8).

(7) $$g^{h}=\sigma \left(F_{h}\left(f^{h}\right)\right)$$

(8) $$g^{w}=\sigma \left(F_{w}\left(f^{w}\right)\right)$$

Finally, a multiplicative weighting calculation is performed on the original feature map, resulting in the final feature map with attention weights in both the height and width directions, as demonstrated in Equation (9).

(9) $$y_{c}\left(i,j\right)=x_{c}\left(i,j\right)\times g_{c}^{h}\left(i\right)\times g_{c}^{w}\left(j\right)$$

In simple terms, CA operates by performing average pooling in both horizontal and vertical directions, followed by a transformation to encode spatial information, and finally integrating the spatial information by weighting it along the channel. This way, the CA module encodes the positional information of images, enabling the network to make better use of positional relationships, further enhancing the quality and accuracy of image dehazing. The introduction of the CA module enables the dehazing network to better understand the spatial structure and positional correlations within images. Based on the weights of pixel coordinates, it adaptively and selectively emphasizes important areas in images, improving the quality and accuracy of image dehazing and offering an effective means to enhance the image dehazing algorithms for underground coal mines.

In summary, this paper constructs a more comprehensive and robust dehazing network by the introduction of CAB into the residual convolution blocks and the integration of the CA module following the feature fusion. CAB captures local information by applying an attention inner patch and captures global information by applying attention between patches on a single-channel feature map. This increases the focus on important regions and details in images, aiding in the more accurate restoration of image details and colors. Meanwhile, the CA module fully utilizes the positional information of pixels, which enables the network to focus more on key areas and edge positions in haze images. This combined attention mechanism enables the dehazing network to analyze and restore the structure and details in images more accurately, thereby enhancing the dehazing performance. In experiments, networks incorporating CBAM and CA modules are evaluated both quantitatively and qualitatively, and the results show that they significantly improve the dehazing performance.

### 3.2. Dynamic Snake Convolution

**Figure 5:**
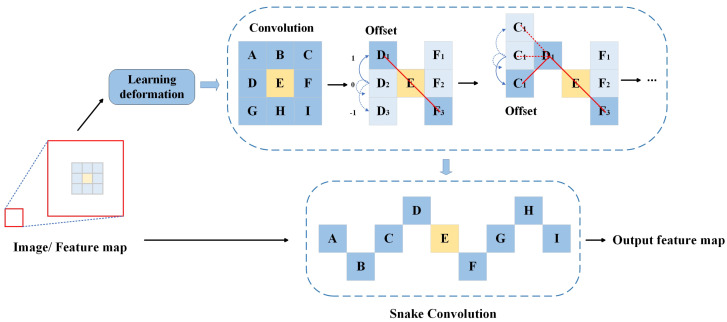
Dynamic Snake Convolution(DSConv), which learns the deformation according to the input feature map, adaptively focuses on the local features of the image.

Due to inadequate illumination and the presence of substantial dust and water haze in coal mines, the images captured by video surveillance systems are often blurry with a significant detail loss. The receptive field of standard convolutions is insufficient to meet the demands of these complex scenes. Moreover, the fixed nature of the standard 3 × 3 convolution kernel limits its ability to capture details, particularly in images with complex backgrounds or rich details, which can lead to information loss and suboptimal feature extraction. Therefore, to better capture and retain more global and detailed information in images, we introduce Dynamic Snake Convolution (DSConv) to replace the first 3 × 3 convolution layer in the encoder part of the network structure, thereby enhancing the feature extraction capability, as illustrated in Figure 5:

DSConv can adaptively focus on the local structures of small objects, enabling the network to better capture these details and reduce information loss. This is crucial for maintaining important details and the overall structural continuity in the image. Additionally, DSConv can adjust the shape and position of the convolution kernel adaptively, allowing it to better handle complex backgrounds, extract more useful information, and reduce background interference, thereby improving the dehazing effect.

For a standard 3 × 3 2D convolution kernel *K*, it is expressed as follows:

(10) K={(x−1,y−1),(x−1,y),⋯,(x+1,y+1)}

DSConv employs an iterative strategy to sequentially select the next position of the target to be processed, thereby ensuring the continuity of attention and preventing the perceptual field from expanding too far due to large deformation offsets. In DSConv, the standard convolution kernel is straightened in both the *x*-axis and *y*-axis directions. Considering a kernel size of 9, the specific position on the *x*-axis *K* is expressed as $K_{i\pm c}=\left(x_{i\pm c},y_{i\pm c}\right)$, where c={0,1,2,3,4} represents the horizontal distance from the central grid. The selection of each grid position $K_{i\pm c}$ in the convolution kernel *K* is a cumulative process. Starting from the central position $K_{i}$, the position farther from the central grid depends on the position of the previous grid. $K_{i+1}$ is augmented with an offset Δ={δ|δ∈[−1,1]} compared with $K_{i}$. Thus, the offset needs to be accumulated to ensure that the convolution kernel conforms to a linear morphological structure. Therefore, the change in the *x*-axis direction is as follows:

(11) $$K_{i\pm c}=\left\{\begin{array}{c} \left(x_{i+c},y_{i+c}\right)=\left(x_{i}+c,y_{i}+\sum_{i}^{i+c}\Delta y\right)\\ \left(x_{i-c},y_{i-c}\right)=\left(x_{i}-c,y_{i}+\sum_{i-c}^{i}\Delta y\right) \end{array}\right.$$

For the *y*-axis direction, the change is as follows:

(12) $$K_{j\pm c}=\left\{\begin{array}{c} \left(x_{j+c},y_{j+c}\right)=\left(x_{j}+\sum_{j}^{j+c}\Delta x,y_{j}+c\right)\\ \left(x_{j-c},y_{j-c}\right)=\left(x_{j}+\sum_{j-c}^{j}\Delta x,y_{j}-c\right) \end{array}\right.$$

Since the offset Δ is usually fractional, while coordinates are generally integers, bilinear interpolation is adopted, which is expressed as follows:

(13) $$K=\sum_{K^{\prime }}B\left(K^{\prime },K\right)\cdot K^{\prime }$$

where *K* represents the fractional locations of Equations (11) and (12), $K^{\prime }$ enumerates all integer spatial locations, and *B* is the bilinear interpolation kernel, which can be decomposed into two one-dimensional kernels as follows:

(14) $$B\left(K,K^{\prime }\right)=b\left(K_{x},K_{x}^{\prime }\right)\cdot b\left(K_{y},K_{y}^{\prime }\right)$$

DSConv enhances the capability to capture slender and tortuous structural features through a series of adaptive adjustment mechanisms, thereby improving the network’s ability to process complex backgrounds and detail-rich images. 

### 3.3. Fusion Loss Function

In image processing tasks, the L1 loss function is commonly used to train networks. Its formula can be represented as follows:

(15) $$L_{L1}\left(x,y\right)=\frac{1}{n}\sum_{i=1}^{n}\left|f\left(x_{i}\right)-y_{i}\right|$$

where $f(x_{i})$ represents the predicted value of the *i*th sample, $y_{i}$ represents the true target value of the *i*th sample, and *n* denotes the number of samples.

Furthermore, the L1 loss function encourages the minimization of the absolute difference between pixel values, so, it can keep the detail information of images in dehazing tasks. In underground coal mines, detail information is crucial for identifying objects, obstacles, and safety issues, and the L1 loss function helps ensure that these details are not blurred during the dehazing process.

However, the L1 loss function only focuses on the differences between pixel values, neglecting how the human visual system perceives images. Additionally, it treats every pixel in images equally, without considering local spatial relationships. Therefore, in the complex environment of the underground coal mines, relying solely on the L1 loss function does not effectively capture the structure of images. To address this, we have incorporated the Multi-Scale Structural Similarity (MS-SSIM) loss function. MS-SSIM extends the Structural Similarity Index Measure (SSIM) by incorporating image information at multiple scales. SSIM measures the similarity between two images based on three comparative measures between the reference image *x* and the evaluated image *y*: luminance, contrast, and structure. The formulas for each component are as follows:

(16) $$l\left(x,y\right)=\frac{2\mu_{x}\mu_{y}+C_{1}}{\mu_{x}^{2}+\mu_{y}^{2}+C_{1}}$$

(17) $$c\left(x,y\right)=\frac{2\sigma_{x}\sigma_{y}+C_{2}}{\sigma_{x}^{2}+\sigma_{y}^{2}+C_{2}}$$

(18) $$s\left(x,y\right)=\frac{\sigma_{xy}+C_{3}}{\sigma_{x}\sigma_{y}+C_{3}}$$

It is commonly set that $C_{3}=C_{2}/2$, so $SSIM\left(x,y\right)={\left[l\left(x,y\right)\right]}^{\alpha }\cdot {\left[c\left(x,y\right)\right]}^{\beta }\cdot {\left[s\left(x,y\right)\right]}^{\gamma }$. Assuming that α, β, and γ are all equal to 1, we can obtain the following:

(19) $$SSIM\left(x,y\right)=\frac{\left(2\mu_{x}\mu_{y}+C_{1}\right)\left(2\sigma_{xy}+C_{2}\right)}{\left(\mu_{x}^{2}+\mu_{y}^{2}+C_{1}\right)\left(\sigma_{x}^{2}+\sigma_{y}^{2}+C_{2}\right)}$$

where *x* and *y* represent the two images, and $\mu_{x}$ and $\mu_{y}$ represent the mean values of images *x* and *y*, respectively. $\sigma_{x}$ and $\sigma_{y}$ represent the standard deviations of images *x* and *y*, $\sigma_{xy}$ represents the covariance between images *x* and *y*, and $C_{1}$ and $C_{2}$ are constants introduced to avoid division by zero.

For a given pixel position *p* in the two images, its SSIM is defined as follows:

(20) SSIM(p)=l(p)·cs(p)

where the calculation of the mean and standard deviation is realized through a Gaussian filter $G_{\sigma_{G}}$ (with $\sigma_{G}$ as its standard deviation). Thus, the SSIM loss function is represented as follows:

(21) $$L_{SSIM}\left(P\right)=\frac{1}{N}\sum_{p\in P}\left(1-SSIM(p)\right)$$

The introduction of MS-SSIM overcomes the issue in SSIM, where $\sigma_{G}$ is artificially set, which affects the image quality. A smaller value of $\sigma_{G}$ might lose the ability to preserve local features, while a larger value may maintain edge noise. If *M* scales are set, then the MS-SSIM formula can be expressed as follows:

(22) $$MS-SSIM\left(p\right)=l_{M}^{\alpha }\left(p\right)\cdot \prod_{j=1}^{M}cs_{j}^{\beta_{j}}\left(p\right)$$

where l(p) and cs(p) are consistent with the SSIM formula. Correspondingly, the MS-SSIM loss function is expressed as follows:

(23) $$L_{\mathrm{MS}-\mathrm{SSIM}}\left(P\right)=\frac{1}{N}\sum_{p\in P}1-\mathrm{MS}-\mathrm{SSIM}\left(p\right)$$

Furthermore, MS-SSIM adopts a nonlinear contrast enhancement function to better simulate the human eye’s perception of image contrast. Compared with SSIM’s linear contrast gain, this nonlinear transformation captures image details and texture features more effectively. By integrating MS-SSIM into our loss function, the dehazed images of underground coal mines visually come closer to the target images, especially in terms of structural textures.

Consequently, to comprehensively consider factors like Structural Similarity, perceptual quality, and detail preservation, a fusion loss function is proposed to train the dehazing network. The loss function is a linear combination of the MS-SSIM loss and the L1 loss, aimed at optimizing the pixel-level accuracy and visual quality in restored images. Specifically, the fusion loss function is defined as follows:

(24) $$L_{Fusion}=\alpha \cdot L_{MS-SSIM}+\beta \cdot L_{L1}$$

where $L_{MS-SSIM}$ represents the Structural Similarity Index Measure loss function, $L_{L1}$ represents the L1 loss function, and α and β are weight coefficients used to balance the importance of the two loss functions.

In summary, this paper introduces a fusion loss function to train the underground coal mines’ dehazing network, taking into account the characteristics of both MS-SSIM and L1 losses. The MS-SSIM loss function considers the Structural Similarity of images, enhancing the ability to preserve the image structure. The L1 loss function is sensitive to image details, enabling better restoration of these details. By linearly combining these two loss functions, we can preserve details while improving the perceptual quality and structural consistency of the images. The fusion loss function overcomes the limitations of MS-SSIM and the L1 losses individually, creating complementary advantages. It balances the enhancement of visual quality driven by MS-SSIM with the pixel-level accuracy promoted by L1 loss. Compared with traditional single-type loss functions, this combination achieves exceptional image dehazing results at both the pixel and structural levels.

## 4. Experiments

### 4.1. Experimental Dataset

This paper employs the public Haze-4K dataset [45] and a self-made underground coal mine dataset for experiments.

The Haze-4K dataset contains 4000 pairs of indoor and outdoor images, divided into a training set and a testing set at a 3:1 ratio. It consists of paired haze and corresponding haze-free images, where each haze image is associated with an underlying clean image, transmission map, and atmospheric light.

**Figure 6:**
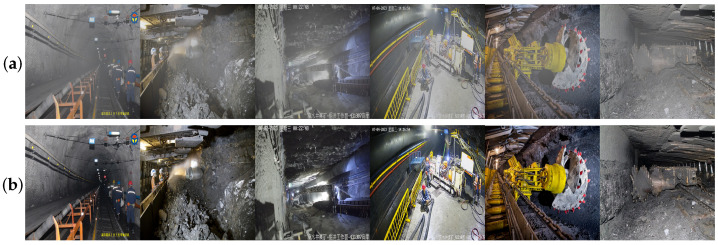
Some image examples from the dataset. (**a**) hazy images in the dataset, (**b**) the corresponding clear, haze-free images.

Additionally, a self-made underground coal mine dataset is used for network training and evaluation. Due to the lack of publicly available underground coal mine datasets, a self-made dataset is created by collecting a large number of images and surveillance videos from various underground coal mines, covering typical underground scenes like conveyor belts, mining faces, and tunnels, across different mining areas and time periods, ensuring the dataset’s diversity and representativeness. It contains 4764 pairs of underground coal mine images in PNG format, each with a resolution of 1024 × 1024, divided into training and testing sets at a 3:1 ratio. Some images from the dataset are shown in Figure 6.

### 4.2. Experimental Configuration

#### 4.2.1. Experimental Environment

The operating system used for this experiment is Windows 10, the Central Processing Unit (CPU) is an Intel(R) Core(TM) i9-9820X CPU @ 3.30 GHz, and the Graphics Processing Unit (GPU) is an NVDIA GeForce RTX 2080Ti. The implementation is carried out using the Python programming language and the deep learning framework PyTorch. The experimental environment is shown in Table 1.

#### 4.2.2. Network Training

In the network training phase, the number of each residual attention convolution block is set to {M,M,M,2M,M,M,M}, and the number of channels is set to {N,2N,4N,8N,4N,2N,N}, with M set to 4 and N set to 24 in this experiment. During the training process, the an AdamW optimizer [46] is used to train the model, with momentum parameters $\beta_{1}$ and $\beta_{2}$ set to 0.9 and 0.999, respectively. Additionally, the cosine annealing strategy [47] is employed to dynamically adjust the learning rate, guiding the model to explore the local optima of the loss function, thereby improving the convergence and generalization ability of the model. The weight decay coefficient is set to 0.01, and to reduce the risk of training collapse, a warmup strategy is introduced with a total of 500 epochs, the first 20 of which are used for warmup. Furthermore, batch training is also utilized, with each batch containing a certain number of image samples, and appropriate training epochs and batch sizes are set to balance the relationship between training speed and model performance. Finally, mixed precision training [48] is employed, allowing low-precision training for certain layers during the training process. This approach reduces computational costs and memory usage without compromising model performance, shortens the training time, and increases the size of mini-batches.

### 4.3. Evaluation Metrics

To objectively and fairly evaluate the dehazing performance of various algorithms, three objective evaluation metrics—Peak Signal-to-Noise Ratio (PSNR), Structural Similarity Index (SSIM), and Mean Squared Error (MSE)—and subjective visual effects are used to assess the performance of algorithms in the task of image dehazing in underground coal mines.

PSNR is a commonly used metric for assessing image quality, evaluating the similarity between the dehazed images and the original images. A higher PSNR value indicates a smaller difference between the dehazed and the original images, suggesting better quality of the dehazed images. The formula for PSNR is as follows:

(25) $$PSNR=10\cdot \log_{10}\left(\frac{{\left(2^{n}-1\right)}^{2}}{MSE}\right)$$

where $\left(2^{n}-1\right)$ is the dynamic range of pixel values. For 8-bit images, $\left(2^{n}-1\right)$ is typically 255, and for 16-bit images, $\left(2^{n}-1\right)$ is 65,535. MSE represents the Mean Squared Error, indicating the difference between pixel values of two images.

SSIM considers differences in luminance, contrast, and structure, enabling it to better capture the structural information of images, particularly for preserving details and textures in reconstructed images. The formula for SSIM is as follows, with its value ranging from 0 to 1. A value closer to 1 indicates that the resulting image is more similar to the original image, implying a higher quality of the images.

(26) $$SSIM\left(x,y\right)=\frac{\left(2\mu_{x}\mu_{y}+C_{1}\right)\left(2\sigma_{xy}+C_{2}\right)}{\left(\mu_{x}^{2}+\mu_{y}^{2}+C_{1}\right)\left(\sigma_{x}^{2}+\sigma_{y}^{2}+C_{2}\right)}$$

where *x* represents the original image; *y* represents the dehazed image; $\mu_{x}$ and $\mu_{y}$ represent the mean values of images *x* and *y*, respectively; $\sigma_{x}$ and $\sigma_{y}$ represent the standard deviations of images *x* and *y*; $\sigma_{xy}$ represents the covariance of pixel values between images *x* and *y*; and $C_{1}$ and $C_{2}$ are constants added for numerical stability.

MSE is a metric used to measure the difference between estimated values and true values. In image dehazing tasks, MSE is used to compare the pixel value differences between the dehazed and the original images. The range of MSE values is typically non-negative, with a lower MSE indicating that the pixel values of the dehazed image are closer to those of the original haze-free image, implying a better dehazing performance. The formula for MSE is as follows:

(27) $$MSE=\frac{1}{N}\sum_{i=1}^{N}{\left(x_{i}-y_{i}\right)}^{2}$$

where $x_{i}$ and $y_{i}$ are the pixel values in the original and dehazed images, respectively, and *N* is the total number of pixels in images.

In addition to the above three objective evaluation metrics, we also integrate subjective visual effects for a comprehensive assessment. It is conducted by directly observing the dehazing results and assessing aspects such as image clarity, detail preservation, contrast, and naturalness. This provides an intuitive personal perception, which is significant for evaluating the algorithm’s performance in practical applications.

By considering PSNR, SSIM, MSE, and subjective visual effects together, this paper comprehensively evaluates the performance of various algorithms in the task of image dehazing in underground coal mines. PSNR measures the noise level, SSIM measures the Structural Similarity, and MSE focuses on the differences in image pixels. The three objective evaluation metrics provide quantitative measures, while the visual effect offers an intuitive subjective assessment, together providing a comprehensive evaluation of the algorithms’ performance.

### 4.4. Experimental Results Analysis

#### 4.4.1. ResAttConv Block and CA Experiment

**Figure 7:**
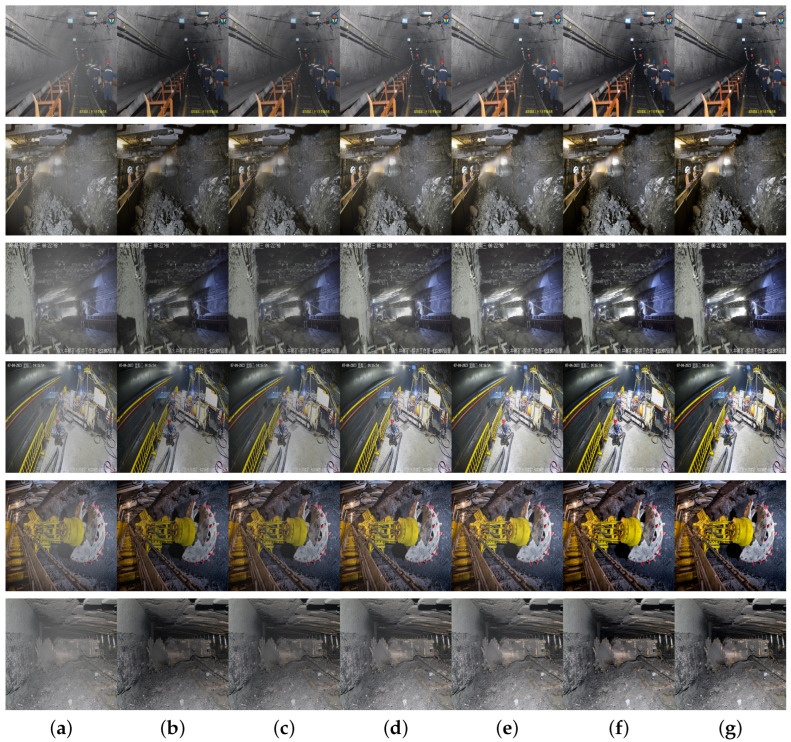
Comparative results of different modules added to the underground coal mine dataset: (**a**) haze image, (**b**) SE module, (**c**) CBAM module, (**d**) CAL module, (**e**) CAB module, (**f**) CA module, and (**g**) CAB + CA modules.

To verify the effectiveness of the Residual Attention Convolution Block and the CA module, the first 3 × 3 convolutional layer in the encoder part of the network structure is replaced with DSConv and the network is trained using the fusion loss function to ensure that, under identical conditions, comparisons and tests are conducted with SE, CBAM, CAL, CAB, and CA modules. The results are illustrated in Figure 7 and Table 2.

The figure above shows the dehazing comparison results of adding different attention modules to the underground coal mine dataset. The dehazed images with the SE module exhibited darkening and haze remnants. The model with the CAL module, due to its high computational complexity, showed a mediocre dehazing performance and color distortion. The dehazing results with the CBAM and CA modules significantly improved, effectively avoiding color distortion, but still had some haze remnants. The dehazing performance with the CAB module was better, while the images processed with the CAB + CA integrated module were clearer, effectively restoring the color and edge texture details of objects under haze.

Table 2 presents a comparison of various metrics with different attention modules added to the underground coal mine dataset. The results show that the method used in this paper achieved the best performance in all metrics. This confirms that the addition of the CAB and CA comprehensive module more effectively dehazes in the underground coal mine environments, and the image structure and texture information are well preserved, resulting in dehazed images with better visual quality.

#### 4.4.2. CNNs Experiment

**Figure 8:**
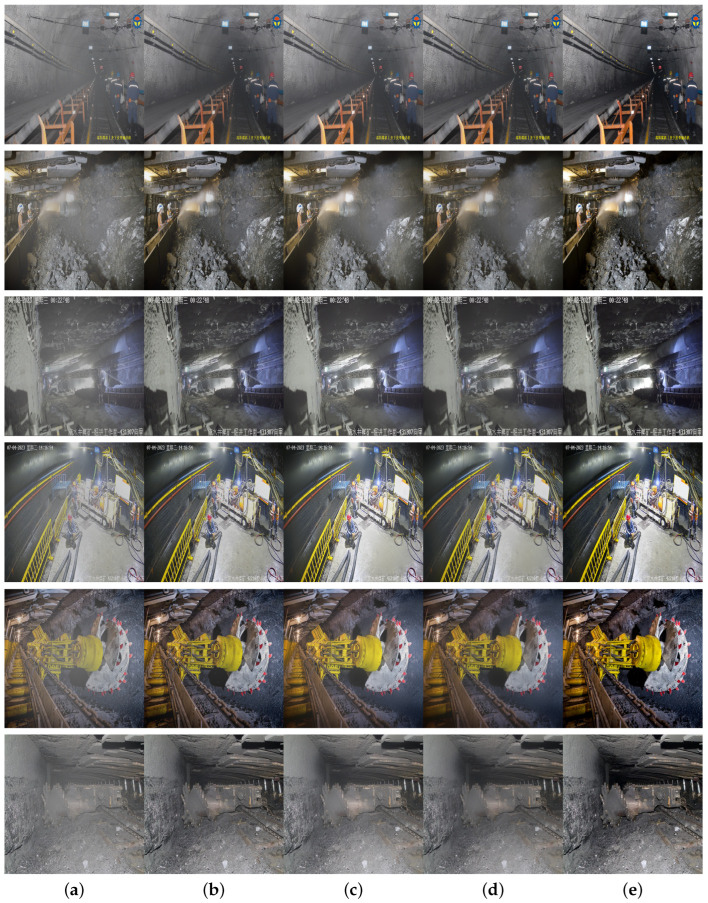
Comparative results of different loss functions used on the underground coal mine dataset: (**a**) haze image, (**b**) DyConv, (**c**) GhostConv, (**d**) DSC, and (**e**) DSConv.

To verify the effectiveness of DSConv, the attention module uses the CAB + CA module, and the network is trained using the fusion loss function. Under identical conditions, comparisons and tests are conducted with DyConv, GhostConv, and DSC. The results are shown in Figure 8 and Table 3.

The comparison results of dehazing with different convolutions on the underground coal mine dataset are shown in Figure 8. As illustrated in the figure, the dehazing effect with DyConv is mediocre, leaving a significant amount of haze. The dehazing effect with GhostConv shows some improvement, but there is a loss of image detail. The dehazing effect with DSC significantly improves, effectively avoiding color distortion. The dehazed images with DSConv are clearer, effectively restoring the color and detail information of objects under haze.

Table 3 presents the comparison results of various metrics using different convolutions on the underground coal mine dataset. The results indicate that the fusion loss function achieved optimal performance across all metrics, confirming that DSConv can more effectively dehaze in the underground coal mine environment. The structural and textural information of the images is well-preserved, and the dehazed images exhibit better visual quality. 

#### 4.4.3. Fusion Loss Function Experiment

**Figure 9:**
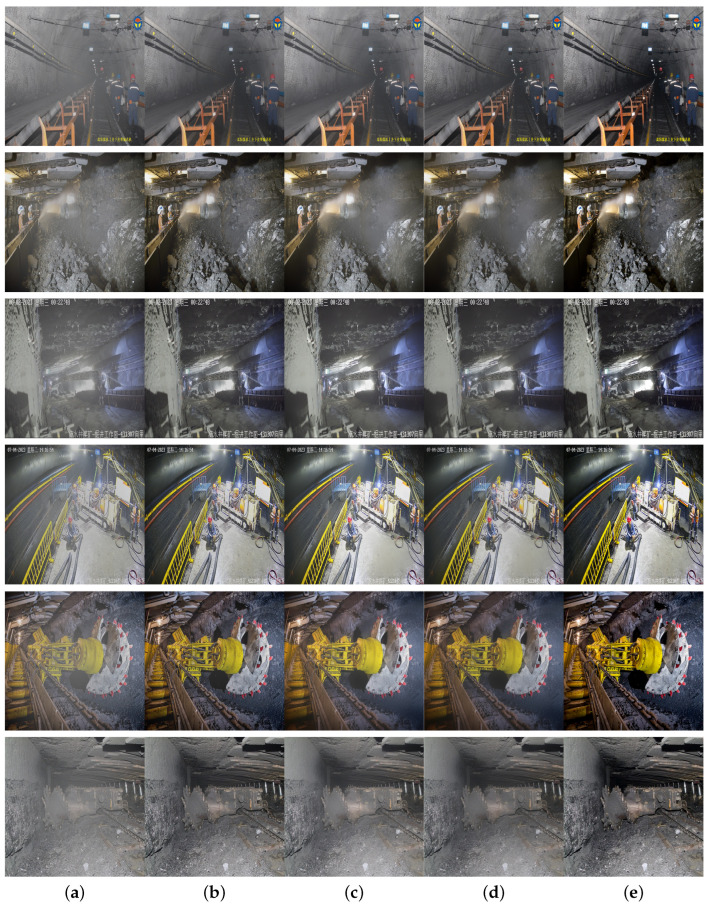
Comparative results of different loss functions used on the underground coal mine dataset: (**a**) haze image, (**b**) L1 loss function, (**c**) SSIM loss function, (**d**) MS-SSIM loss function, and (**e**) fusion loss function.

To verify the effectiveness of the fusion loss function, the attention module utilizes the CAB + CA module, and the first 3 × 3 convolutional layer in the encoder part of the network structure is replaced with DSConv. Under identical conditions, comparisons and tests are conducted with the L1 loss function, SSIM loss function, and MS-SSIM loss function. The results are illustrated in Figure 9 and Table 4.

The figure above shows the dehazing comparison results of using different loss functions with the underground coal mine dataset. As seen from Figure 9, the L1 loss function fails to effectively capture the structural information of images, while the SSIM and MS-SSIM loss functions show a poor ability to restore details. The fusion loss function, however, can effectively restore the detail textures and structural information of objects under the haze.

Table 4 presents a comparison of various metrics using different loss functions with the underground coal mine dataset. The results demonstrate that the fusion loss function achieves optimal performance in all metrics. This confirms that the fusion loss function is more effective in dehazing in underground coal mine environments, preserving the structure and texture information of images well, resulting in dehazed images with better visual quality.

#### 4.4.4. CCDF-gUNet Experiment

**Figure 10:**
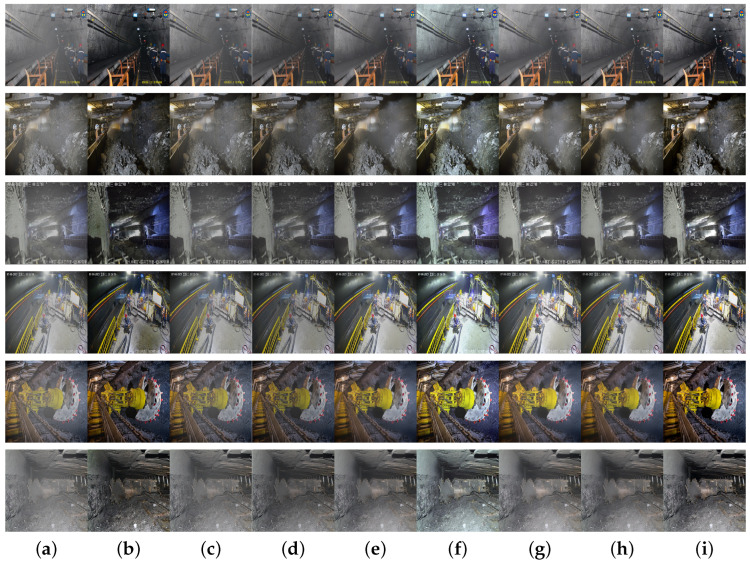
Subjective visual effects of different algorithms on the underground coal mine dataset: (**a**) haze image, (**b**) DCP algorithm, (**c**) AOD-Net algorithm, (**d**) FFA-Net algorithm, (**e**) gUNet algorithm, (**f**) DehazeFormer algorithm, (**g**) RIDCP algorithm, (**h**) SFSNiD algorithm, and (**i**) ours.

To verify the dehazing effect of the algorithm proposed in this paper in a real underground coal mine environment, we compare and analyze the experimental results with DCP, AOD-Net, FFA-Net, gUNet, RIDCP, and SFSNiD algorithms under the same configuration conditions. Six randomly selected dehazing images from the underground coal mine dataset are used for subjective visual comparative analysis, as shown in Figure 10.

As can be observed from the picture above, the images processed with DCP exhibit an overall darkening effect, accompanied by color distortion. The AOD-Net algorithm leaves a significant amount of haze in the processed images, leading to suboptimal dehazing effects. Images processed with the FFA-Net algorithm show localized darkening and inadequate detail restoration capabilities. Both gUNet and DehazeFormer demonstrate better dehazing effects, but images processed with gUNet exhibit loss of details and textures, while those processed by DehazeFormer come with color distortion. The dehazing performance of RIDCP in handling non-uniform haze scenarios in underground coal mines is suboptimal. Although SFSNiD demonstrates superior dehazing capabilities, its model running time is longer. In contrast, the algorithm proposed in this paper outperforms the other methods in dehazing effectiveness, effectively restoring the color and detailed textures of objects under the haze, preserving the color and structure of the images, and visually resembling clear, haze-free images more closely, and the algorithm operates with a shorter running time.

For a more objective and impartial evaluation of the dehazing performance of the above algorithms, tests are conducted on the public dataset Haze-4K and the self-made underground coal mine dataset under the same configuration conditions. PSNR, SSIM, and MSE are selected as objective evaluation metrics, and their average values are used for comparative analysis. The results of the objective evaluation metrics are presented in Table 5.

As shown in Table 5, the proposed algorithm does not achieve the best performance on the public Haze-4K dataset because it is specifically designed for the complex scenarios of underground coal mines, which may not be applicable to normal scenes. However, the algorithm demonstrates superior dehazing performance on the underground coal mine dataset. Compared with other algorithms, it achieves the best results across all three metrics, indicating its excellent dehazing capability and suitability for the complex conditions in underground coal mines.

#### 4.4.5. Ablation Study

Additionally, to verify the effectiveness of each module in the dehazing process, an ablation study is designed. In this study, Model 1 represents the original gUNet model; Model 2 represents the gUNet model with the addition of the CAB + CA module; Model 3 represents the gUNet model with the addition of the DSConv module; Model 4 represents the gUNet model with the incorporation of the fusion loss function for network training; Model 5 represents the gUNet model with the addition of both the CAB + CA and DSConv modules; Model 6 represents the gUNet model with the addition of the CAB + CA module and the incorporation of the fusion loss function for network training; Model 7 represents the gUNet model with the addition of the DSConv module and the incorporation of the fusion loss function for network training; and Model 8 represents the gUNet model with the addition of both the CAB + CA and DSConv modules, as well as the incorporation of the fusion loss function for network training, which is the network proposed in this paper. The ablation study on the self-made underground coal mine dataset is conducted for this dehazing model, calculating the PSNR and SSIM between the output dehazed images and the original hazy images for comparative analysis, as shown in Table 6. The table clearly demonstrates the effectiveness of each module.

## 5. Conclusions

This paper proposes a novel image dehazing algorithm for underground coal mine environments, named CCDF-gUNet. Initially, to reduce the number of parameters, a lightweight U-Net model is employed as the basic architecture to extract multi-scale information from images, and DSConv is introduced to replace traditional convolutions, enhancing the feature extraction capability. Subsequently, residual attention convolutional blocks and CA modules are used to enhance the model’s ability to perceive significant features, enabling a better capture of key information in images. Finally, a fusion loss function is introduced to train the dehazing model, simultaneously focusing on the details and structural consistency of the images. Extensive experiments conducted on the public dataset and a self-made underground coal mine dataset demonstrate that the proposed algorithm effectively avoids color distortion in subjective visual effects and preserves details and the edge information of images to a certain extent. It also achieves favorable results in objective evaluation metrics, providing a theoretical reference for image processing in coal mine monitoring videos.

While the CCDF-gUNet algorithm proposed in this paper achieves commendable dehazing results in underground coal mine environments, it still has certain limitations. The model does not currently consider the low-light conditions prevalent in underground coal mines. In the future, we will further optimize this network model to achieve effective dehazing in low-light environments.

## Figures and Tables

**Table 1:** Experimental environment.

Configuration	Parameter
operating system	Windows 10
CPU	Intel(R) Core(TM) i9-9820X CPU @ 3.30 GHz
GPU	NVIDIA GeForce RTX 2080Ti
deep learning framework	PyTorch

**Table 2:** Quantitative comparison of different attention modules added to the underground coal mine dataset.

Module	PSNR/dB	SSIM	MSE
SE	25.02	0.936	204.25
CBAM	26.43	0.949	148.34
CAL	25.38	0.937	187.53
CAB	28.02	0.961	102.14
CA	27.94	0.957	104.08
CAB + CA	31.18	0.971	49.66

**Table 3:** Quantitative comparison of different convolutions added to the underground coal mine dataset.

Convolutions	PSNR/dB	SSIM	MSE
DyConv	25.23	0.937	193.53
GhostConv	26.06	0.941	160.61
DSC	27.57	0.951	112.93
DSConv	31.18	0.971	49.66

**Table 4:** Quantitative comparison of different loss functions used on the underground coal mine dataset.

Module	PSNR/dB	SSIM	MSE
L1	24.82	0.931	214.95
SSIM	25.96	0.936	161.02
MS-SSIM	27.21	0.947	123.23
Fusion	31.18	0.971	49.66

**Table 5:** Results of objective evaluation metrics for different algorithms on Haze-4K and the underground coal mine dataset.

Algorithm	Haze-4K Dataset			Underground Coal Mine Dataset		
	PSNR/dB	SSIM	MSE	PSNR/dB	SSIM	MSE
DCP	18.17	0.796	785.49	17.66	0.739	894.74
AOD-Net	20.90	0.911	576.58	19.50	0.851	645.07
FFA-Net	23.26	0.932	306.36	23.34	0.919	303.03
gUNet	24.06	0.927	256.62	24.03	0.925	259.02
DehazeFormer	25.45	0.935	162.42	25.32	0.931	180.44
RIDCP	30.86	0.978	53.28	27.53	0.943	114.28
SFSNiD	32.17	0.973	39.38	28.66	0.958	87.92
Ours	30.72	0.976	55.04	31.18	0.971	49.66

**Table 6:** Ablation study results.

Model	1	2	3	4	5	6	7	8
baseline	✓	✓	✓	✓	✓	✓	✓	✓
CAB + CA	✗	✓	✗	✗	✓	✓	✗	✓
DSConv	✗	✗	✓	✗	✓	✗	✓	✓
Fusion loss function	✗	✗	✗	✓	✗	✓	✓	✓
PSNR	24.03	25.84	26.36	26.91	27.54	28.05	27.83	31.18
SSIM	0.925	0.933	0.935	0.939	0.948	0.953	0.956	0.971

## Data Availability

The raw data supporting the conclusions of this article will be made available by the authors on request.

## Abbreviations

The following abbreviations are used in this manuscript:

DSConv	Dynamic Snake Convolution
HQPs	high-quality priors
SFSNiD	spatial and frequency domain-aware semi-supervised nighttime dehazing network
SFII	spatial and frequency domain information interaction
CAL	Counterfactual Attention Learning
CAB	Cross Attention Block
IPSA	Inner-Patch Self-Attention Block
CPSA	Cross-Patch Self-Attention Block
DyConv	dynamic convolution
GhostConv	ghost convolution
DSC	depthwise separable convolution
LN	Layer Normalization
MSA	Multi-Head Self-Attention
CBAM	Convolutional Block Attention Module
CA	Coordinate Attention
PSNR	Peak Signal-to-Noise Ratio
SSIM	Structural Similarity
MSE	Mean Squared Error
ASM	Atmospheric Scattering Model
DCP	Dark Channel Prior
CNN	Convolutional Neural Network
AOD-Net	All-in-One Dehazing Network
LD-Net	Light-DehazeNet
FFA-Net	Feature Fusion Attention Network
MSBDN	Multi-Scale Boosted Dehazing Network
STN	Spatial Transformer Network
SE-Net	Squeeze-and-Excitation
MS-SSIM	Multi-Scale Structural Similarity
CAM	Channel Attention Module
SAM	Spatial Attention Module
MLP	Multi-Layer Perceptron
FC	fully connected
CPU	Central Processing Unit
GPU	Graphics Processing Unit
